# Genome sequences of *Verticillium longisporum* strains M058 and M219, members of the A1D1 type-II lineage

**DOI:** 10.1128/mra.00168-26

**Published:** 2026-04-27

**Authors:** Annette Pfordt, Dominic Simm, Birger Koopmann, Andreas von Tiedemann, Martin Kollmar

**Affiliations:** 1 Plant Pathology and Crop Protection Division, Department of Crop Sciences, Georg-August-University Göttingen, Göttingen, Germany; 2 GOENOMICS GmbH, Göttingen, Germany; University of Maryland School of Medicine, Baltimore, Maryland, USA

**Keywords:** genome sequences, *Verticillium longisporum*

## Abstract

We report chromosome-scale genome assemblies and annotations of two *Verticillium longisporum* A1D1 type-II strains, M058 and M219, isolated from oilseed rape in the United Kingdom and Germany. These high-quality genomes provide resources for studying hybrid genome evolution, host adaptation, and pathogenicity in *Brassica*-infecting populations.

## ANNOUNCEMENT

*Verticillium* species are soilborne ascomycete fungi that colonize plant xylem and include major vascular wilt pathogens with pronounced host specificity. *Verticillium longisporum* is an interspecific hybrid species that primarily infects Brassicaceae, including oilseed rape (*Brassica napus*). Based on phylogenetic and population genomic analyses, *V. longisporum* isolates are classified into three main hybrid lineages—A1D1, A1D2, and A1D3—which differ in host range, geographic distribution, and virulence traits (1, 2). The A1D1 lineage is further subdivided into type-I to type-IV subgroups (3), which are genetically and epidemiologically distinct and dominate oilseed rape infections in Europe (2, 4).

Here, we report the genome assemblies and annotations of two *V. longisporum* A1D1 type-II strains, M058 and M219. M058 was isolated in 2014 from oilseed rape in Wickhambrock, United Kingdom, whereas M219 was isolated in 2016 from oilseed rape in Schöningen, Germany. Both strains represent agriculturally relevant European populations and provide valuable resources for comparative and functional genomics within the A1D1 type-II subgroup. Infected oilseed rape tissues were surface-sterilized and placed on potato dextrose agar medium. After 14 days of incubation at 22°C, colonies of *V. longisporum* were obtained, and single-spore cultures were established for further analyses.

DNA extraction and sequencing were performed by BMKGene (Münster, Germany). HMW genomic DNA was extracted from 0.5 g of mycelial tissue using a CTAB-based extraction method. In this protocol, sodium acetate was used instead of sodium chloride; it was omitted from the extraction buffer and applied during the precipitation step. For library preparation, 0.75–1.5 μg of genomic DNA was mechanically sheared using g-TUBE devices and subsequently purified with AMPure PB beads according to the manufacturer’s protocol. No size selection was applied. Sequencing libraries were generated using SMRTbell Prep Kit 3.0 and sequenced on the PacBio Revio (Table 1). Reads were assembled using hifiasm v0.25.0-r726 (5) using parameters --primary -l0 (default parameters were used except where otherwise noted), producing chromosome-scale draft assemblies. The NCBI Foreign Contamination Screen v0.5.5 (6) was used to detect contaminations. A representative mitochondrial contig was generated with mitohifi v3.2.1 (7).

**TABLE 1:** Assembly and annotation statistics for *Verticillium longisporum* strains M058 and M219

Statistical item	M058	M219
Total number of raw reads	639,647	560,515
Total read bases (bp)	3,893,094,311	3,429,996,241
Reads N50 (bp)	7,423	7,715
Total assembly length (bp)	73,401,519	73,332,716
Number of contigs	46	34
Number of contigs >1 Mb	16	17
Number of contigs <50 kb	26	16
Mitochondrial contigs	1	1
N50 (Mb)	5.19	4.69
Largest contig (Mb)	8.02	8.02
GC content (%)	52.76	52.76
Estimated coverage (×)	49	44
BUSCO completeness (%)	99.2	99.2
Predicted protein-coding genes	19,361	19,367
Total gene models	22,488	22,539

The assembled genome sizes were approximately 73.40 and 73.33 Mb, with GC contents of 52.76% and 52.76%, respectively (Table 1). Assembly contiguity was consistent with chromosome-scale assemblies, with N50 values of 5.19 Mb for M058 and 4.69 Mb for M219. One of the chromosomes of M219 is split into two parts, based on alignment with the M058 assembly, suggesting 16 chromosomes for A1D1 type-II species. Thirteen (M058) and 12 (M219) of the non-chromosome contigs contain ITS regions.

Genome completeness was assessed using BUSCO (8) with the glomerellales_odb12 data set, yielding completeness scores of 99.2% for M058 and 99.2% for M219 (single and duplicates/multiples). Structural gene annotation was performed using a combination of homology-based prediction, transcript evidence, and ab initio modeling with the Mendle-analytics pipeline, resulting in 19,361 (M058) and 19,367 (M219) predicted protein-coding genes.

These genome resources expand the genomic representation of *V. longisporum* A1D1 type-II strains and will support studies on hybrid genome evolution, host adaptation, and pathogenicity in *Brassica* crops.

## Data Availability

These whole genome shotgun projects have been deposited at DDBJ/ENA/GenBank under accession numbers JBSUOK000000000 (M058) and JBSUOL000000000 (M219). The versions described in this paper are JBSUOK010000000 and JBSUOL010000000. The BioProject can be found under accession number PRJNA1375490 and the SRA under accession numbers SRR36998059 (M058) and SRR36998060 (M219). Annotation is available via Figshare (https://doi.org/10.6084/m9.figshare.31655614).

## References

[1] Inderbitzin P, Davis RM, Bostock RM, Subbarao KV. 2011. The ascomycete Verticillium longisporum is a hybrid and a plant pathogen with an expanded host range. PLoS One 6:e18260. doi:10.1371/journal.pone.0018260 21455321 PMC3063834

[2] Inderbitzin P, Subbarao KV. 2014. Verticillium systematics and evolution: how confusion impedes Verticillium wilt management and how to resolve it. Phytopathology 104:564–574. doi:10.1094/PHYTO-11-13-0315-IA 24548214

[3] Vega‐Marin M, Obermeier C, Koopmann B, Zheng X, Snowdon R, von Tiedemann A. 2025. Phenotypic and phylogenetic analysis of Verticillium longisporum strains from European and Canadian oilseed rape fields. Plant Pathol 74:196–209. doi:10.1111/ppa.14009

[4] Depotter JRL, Deketelaere S, Inderbitzin P, Tiedemann AV, Höfte M, Subbarao KV, Wood TA, Thomma BPHJ. 2016. Verticillium longisporum, the invisible threat to oilseed rape and other brassicaceous plant hosts. Mol Plant Pathol 17:1004–1016. doi:10.1111/mpp.12350 26663851 PMC6638321

[5] Cheng H, Concepcion GT, Feng X, Zhang H, Li H. 2021. Haplotype-resolved de novo assembly using phased assembly graphs with hifiasm. Nat Methods 18:170–175. doi:10.1038/s41592-020-01056-5 33526886 PMC7961889

[6] Astashyn A, Tvedte ES, Sweeney D, Sapojnikov V, Bouk N, Joukov V, Mozes E, Strope PK, Sylla PM, Wagner L, Bidwell SL, Brown LC, Clark K, Davis EW, Smith-White B, Hlavina W, Pruitt KD, Schneider VA, Murphy TD. 2024. Rapid and sensitive detection of genome contamination at scale with FCS-GX. Genome Biol 25:60. doi:10.1186/s13059-024-03198-7 38409096 PMC10898089

[7] Uliano-Silva M, Ferreira JGRN, Krasheninnikova K, Darwin Tree of Life Consortium, Formenti G, Abueg L, Torrance J, Myers EW, Durbin R, Blaxter M, McCarthy SA. 2023. MitoHiFi: a python pipeline for mitochondrial genome assembly from PacBio high fidelity reads. BMC Bioinformatics 24:288. doi:10.1186/s12859-023-05385-y 37464285 PMC10354987

[8] Tegenfeldt F, Kuznetsov D, Manni M, Berkeley M, Zdobnov EM, Kriventseva EV. 2025. OrthoDB and BUSCO update: annotation of orthologs with wider sampling of genomes. Nucleic Acids Res 53:D516–D522. doi:10.1093/nar/gkae987 39535043 PMC11701741

